# Coal-Miners' Nystagmus

**Published:** 1908-10-17

**Authors:** 


### Medicine.

### COAL-MINERS' NYSTAGMUS.

The remarks of Mr. Simeon Snell, in his Presidential address before the British Medical Association, are full of interest, in that they draw attention to the comparative commonness of nystagmus amongst coal-miners, and in that they lay stress upon the fact that this nystagmus is no mere pathological curiosity, but a matter of the very gravest importance both to the employers and to the employees in coal-pits.

To the employers the importance is twofold; in the first place the nystagmus of one man may cause extensive damage to the other miners and to the mine itself by reason of explosions; in the second, the employee who develops nystagmus as the result of his work has a good claim for personal compensation. It is really unsafe to allow a miner with nystagmus to continue his work, and the only cure for the trouble is complete cessation of the work. The employer, therefore, must either compensate the man for complete disablement, or else find him other employment.

The affection is at first slight, and it appears to be slowly progressive over years until giddiness, great difficulty in seeing things in the dusk (night blindness), and a subjective sensation as if everything looked at were oscillating, become extremely troublesome. In respect to the latter symptom, miner's nystagmus differs essentially from some other forms of nystagmus, particularly the congenital form; and it is on this account that the condition is so dangerous. And for this reason: One of the first signs of there being an excess of the explosive fire-damp in the atmosphere of the mine is the development of a blue cap to the flame of the Davy lamp. This blue cap, at first quite small, gradually becomes taller and taller as the percentage of fire-damp in the air increases.

The blue cap on the Davy lamp flame is a danger signal which needs to be reported immediately; otherwise, as the percentage of fire-damp rises, an explosion becomes more and more imminent; even short of the explosion the atmosphere is a very unhealthy one to breathe. In a man whose eyes are constantly oscillating from side to side and whose retinal and cerebral images of the Davy lamp flame and its blue cap are oscillating in the same way, the result is such a blurring of the whole picture that no blue cap can be seen until it is very large indeed—that is to say, until the percentage of fire-damp is already more than dangerous. Mr. Snell has devised a method of illustrating the case very graphically indeed.

He fixed a Davy lamp, alight, and focussed two photographic cameras upon it; the one camera was fixed and still, the other was made to oscillate through a distance of less than half an inch, at the rate of 150 times per minute; the clear picture taken by the first camera and the blurred picture taken by the other were exhibited side by side, the former representing the image of the lamp and flame as seen by a normal person, the latter giving some idea of the same lamp and flame as seen by the nystagmic miner. It is no theoretical picture. A whole series of miners with nystagmus were tested; some could not detect the warning fire-damp flame at all, whilst most of them could only see it when the blue cap was already large.

As to how the nystagmus comes about there is little or no real knowledge; one hopes that one of the results of Mr. Snell's address may be that physiological and pathological experiments and researches will be carried out with a view to determining its cause. Many different theories have been propounded, most of which go little further than attributing it to "occupation," and comparing it to writer's cramp and other occupation neuroses. It is true the visual acuity is nearly always little impaired, if at all, and there are no organic changes to be detected in the eye by the ophthalmoscope; but there are many features of the condition which render it much more like an organic disease than a neurosis.

Most important of all these, perhaps, is the fact that it is not every kind of miner who is liable to the affection. Indeed, it is confined, or practically confined, to coal-miners. Miners of tin and copper, phosphate-miners, ore-miners of various kinds, may work in equally dark places, and for equally long hours in equally cramped positions, without developing nystagmus at all. This is a very significant fact, and one upon which too little stress is laid as a rule. It is often spoken of as "miner's nystagmus"—it should be distinctly designated "coal-miner's nystagmus." It would seem, therefore, that the usual account of its being due to work in a cramped position, necessitating the use of the arms above the head, with a sideways position of the latter, is not really essential.

Imperfections of the light in which the miner works have been accused of being a factor in its causation; if this be true, the factor can only be a very minor one, for the light is often very bad in mines other than coal-mines; and in coal-mines the incidence of nystagmus bears no inverse ratio to the goodness of the lighting.

One would think that histological investigations of the cerebellar cells, and those of the nuclei of the various nerves that control the eye-movements, might throw some light upon the nature of the disease. One cannot help feeling that there must be changes produced that should be recognisable under the microscope; by analogy with cerebellar tumours, and with disseminated sclerosis some slowly progressive organic changes seem likely to be present. Further, one must try to explain why coal-miners should suffer and other miners not. One remembers that fire-damp and other allied gases occur in coal-mines and not in others. One would not be surprised to hear that the long-continued action of small quantities of fire-damp was enough to cause, at any rate in a certain proportion of cases, slow degenerations in the cerebellum, or pons, or corpora quadrigemina.

